# The Efficacy and Safety of Current Treatments in Diabetic Macular Edema: A Systematic Review and Network Meta-Analysis

**DOI:** 10.1371/journal.pone.0159553

**Published:** 2016-07-19

**Authors:** Lu Zhang, Wen Wang, Yan Gao, Jie Lan, Lixin Xie

**Affiliations:** 1 Department of Ophthalmology, School of Medicine, Shandong University, Jinan, China; 2 State Key Laboratory Cultivation Base, Shandong Provincial Key Laboratory of Ophthalmology, Shandong Eye Institute, Shandong Academy of Medical Sciences, Qingdao, China; 3 Department of Neurosurgery, The Second Affiliated Hospital of Soochow University, Suzhou, China; Queen's University Belfast, UNITED KINGDOM

## Abstract

**Purpose:**

To compare the efficacy and safety of current treatments in diabetic macular edema (DME).

**Methods:**

PubMed, Embase and CENTRAL were systematically reviewed for randomized controlled trials of current treatments in DME through August 2015. Data on the mean change of best-corrected visual acuity (BCVA) and central macular thickness (CMT) were extracted, and adverse events (AEs) were collected.

**Results:**

A total of 21 trials were included in our network meta-analysis. Intravitreal ranibizumab improved BCVA most significantly (OR: +7.01 95%CI (2.56 to 11.39)) in 6 months and intravitreal aflibercept (+8.19 (5.07 to 11.96)) in 12 months. Intravitreal triamcinolone combined with LASER decreased CMT most significantly (-111.34 (-254.61 to 37.93)) in 6 months and intravitreal aflibercept (-110.83 (-190.25 to -35.27)) in 12 months. Compared with the relatively high rate of ocular AEs in the groups with administration of steroids, systematic AEs occurred more frequently in the groups with vascular endothelial growth factor inhibitors involved.

**Conclusions:**

Our analysis confirms that intravitreal aflibercept is most favorable with both BCVA improvement and CMT decrease than other current therapies in the management of DME within 12 months. Vascular endothelial growth factor inhibitors for DME should be used with caution due to systematic AEs. Combined intravitreal triamcinolone with LASER has a stronger efficacy in decreasing CMT than the other interventions in the early stage after injection. More high-quality randomized controlled trials will be necessary.

## Introduction

Diabetic macular edema (DME) is a common manifestation of diabetic retinopathy and a leading cause of serious central visual loss and impairment in diabetic patients [1]. The prevalence rate of DME has been reported to be 29% in patients with a duration of more than 20 years [2]. The chance of spontaneous improvement in the best-corrected visual acuity (BCVA) and decrease in the central macular thickness (CMT) is limited, so the prognosis of DME is generally disappointing.

Since the Early Treatment Diabetic Retinopathy Study (ETDRS) found that laser resulted in a 50% reduction in severe vision loss in 1985, laser has been the gold standard treatment for macular edema [3]. Recently, it was reported that the level of inflammatory factors could be significantly elevated in the vitreous fluid of patients with DME, and vascular endothelial growth factors (VEGFs) had a stronger influence on retinal vascular permeability and severity of DME than the other factors [4–7]. To avoid the ocular side effects related to laser treatment like subretinal fibrosis and laser scars [8], the anti-inflammatory drugs, like steroids and VEGF inhibitors, were used for DME by intravitreal injection.

Steroids are the common, anti-inflammatory, anti-angiogenic, and blood-retinal barriers, which can stabilize medications in the treatment of DME. High rates of intraocular pressure (IOP) increase and cataract progress were found in steroids-treated eyes. In recent years, the introduction of VEGF inhibitors has revolutionized the treatment of DME. Bevacizumab, ranibizumab, pegaptanib and alibercept have been well established via phase II/III clinical trials showing significantly improved vision in many patients with DME [9–13]. Bevacizumab, a humanized full-length monoclonal antibody, inhibits all isoforms of VEGFs. It has been popular because it is more affordable than other anti-VEGF drugs in the treatment of retinal vascular diseases [14]. As a monoclonal antibody, ranibizumab blocks all isoforms of VEGF-A and enhances their affinity. The FDA has approved it for the treatment of DME [15]. Pegaptanib can inhibit VEGF-165 responding for ocular neovascularization and vascular permeability [16], and the FDA has approved it for neovascular age-related macular degeneration [17]. Aflibercept is a soluble protein and blocks all isoforms of VEGFs. Its half-life is prolonged, and its affinity of binding to VEGF-A is more than 100-fold greater than bevacizumab or ranibizumab. Anti-VEGF agents must be given frequently, and this may be associated with a small increased risk of systematic adverse effects like hypertension.

Some researchers performed systematic reviews and standard meta-analyses on therapies of DME [18–20]. However, standard meta-analysis is unable to include all direct and indirect comparisons among steroids, VEGF inhibitors, and LASER which included laser, macular laser, grid laser and focal/grid laser in one analysis, while network meta-analysis can conduct a more accurate ranking and precision for the current therapeutic strategies [21–23]. Therefore, we performed a network meta-analysis to estimate the efficacy and safety of current treatments in DME.

## Methods

### Search Strategy

We searched and identified the relevant trials from PubMed, Embase and CENTRAL through August 2015 with no language limit. The medical subject heading and keywords used for the search included *diabetic macular edema*, *laser*, *steroids*, *vascular endothelial growth factor*, *bevacizumab*, *ranibizumab*, *pegaptanib and aflibercept*. We also screened the reference lists of published meta-analyses of DME treatment. BCVA, CMT, and ocular and systematic adverse events (AEs) were the primary endpoints of this meta-analysis.

### Eligibility of Studies

All included studies met the following criteria: (a) randomized controlled trials (RCTs); (b) participants with any type of diabetes; (c) follow-up of more than six months; (d) efficacy outcomes including BCVA and CMT; (e) only the articles containing complete data were included, and duplicated publications or reports of one trial were included once.

Investigators extracted data independently and made the final selection based on resolved discrepancies by discussion. A total of 21 trials were included in our network meta-analysis (Table 1).

**Table 1:** Study characteristics of trials included in the network meta-analysis.

Study ID	Diabetes Mellitus		Treatment			Follow-up	Total (eyes)	BCVA-6m	BCVA-12m	CMT-6m	CMT-12m	Jadad
	Duration (years)	Type 2 (%)	Intervention	Dosage	Number							
Wells et al. (2015)	15±3.32	88%	IVA	2.0mg	9–10	12 months	N = 208	NA	+13.3 ± 11.1	NA	-169 ± 138	4
	17±3.32	94%	IVB	1.25mg	9–11		N = 206	NA	+9.7 ± 10.1	NA	-101 ± 121	
	16±3.06	90%	IVR	0.3mg	9–11		N = 206	NA	+11.2 ± 9.4	NA	-147 ± 134	
Ishibashi et al. (2015)	11.21±8.20	99.2%	IVR	0.5mg	7.8±2.94	12 months	N = 133	NA	+6.6 ± 7.68	NA	-134.6 ± 131.17	7
	11.33±8.05	98.5%	IVR+LASER	0.5mg	7.0±3.07		N = 132	NA	+6.4 ± 10.67	NA	-171.8 ± 160.85	
					1.5±0.85							
	11.34±8.85	98.5%	LASER	NA	1.9±1.02		N = 131	NA	+1.8 ± 8.27	NA	-57.2 ± 118.60	
Berger et al. (2015)	16.5±9.0	88%	IVR	0.5mg	9.2±2.8	12 months	N = 75	+7.1 ± 7.83	+8.9 ± 7.83	–129.3 ± 118.69	–143.5 ± 148.25	5
	18.5±11.6	79.5%	IVR+LASER	0.5mg	8.8±2.9		N = 73	+5.6 ± 8.58	+8.2 ± 9.44	–114.2 ± 113.29	–152.2 ± 142.47	
					1.6±1.0							
	16.6±10.7	87.5%	LASER	NA	2.6±2.1		N = 72	+0.9 ± 7.68	+0.3 ± 13.64	–64.4 ± 117.26	–107.1 ± 157.34	
Gillies et al. (2014)	16.7±10.7	NA	IVB	1.25mg	8.6	12 months	N = 42	NA	+8.9 ± 8.8	NA	NA	5
	16.7±10.3	NA	DDSI	0.7mg	2.7		N = 46	NA	+5.6 ± 16.3	NA	NA	
Comyn et al. (2014)	18.5±4.09	81.8%	IVR	0.5mg	9.0	48 weeks	N = 22	NA	NA	NA	-131.5 ± 98.0	5
	18±2.81	100%	LASER	NA	2.6		N = 11	NA	NA	NA	-102.9 ± 88.4	
Shoeibi et al. (2013)	NA	NA	IVB	1.25mg	1 (1–2)	13.3 ± 3.4 months	N = 41	NA	+14±16	NA	-91 ± 224.9	7
	NA	NA	IVB+IVT	1.25mg	1 (1–2)		N = 37	NA	+9.5±16.5	NA	-57 ± 225	
				2mg	1							
Nepomuceno et al. (2013)	15.9±8.0	NA	IVR	0.5mg	7.67	48 weeks	N = 28	NA	+14.5±2	NA	-136 ± 23	7
	16.2±8.0	NA	IVB	1.5mg	9.84		N = 32	NA	+11.5±1	NA	-126 ± 25	
Callanan et al. (2013)	NA	NA	DDSI+LASER	0.7mg	2	12 months	N = 126	NA	+2.9 ± 11.45	NA	-102.8 ± 130.86	7
					≥1							
	NA	NA	LASER	NA	2.5		N = 127	NA	+2.1 ± 12.05	NA	-125.3 ± 123.38	
Arevalo et al. (2013)	NA	NA	IVB	1.25 or 2.5mg	5.8±3.2	24 months	N = 141	+8.16 ± 17.37	+11.13 ± 17.65	NA	NA	5
	NA	NA	LASER	NA	2.2±1.4		N = 120	+4.1 ± 11.3	+4.41 ± 11.7	NA	NA	
	NA	NA	IVB+LASER	1.25 or 2.5mg	6.2±4.9		N = 157	+4.9 ± 16.37	+7.44 ± 16.73	NA	NA	
Berger et al. (2013)	17±10.1	86%	IVR+LASER	0.5mg	Monthly	12 months	N = 78	NA	+8.0 ± 9.1	NA	-145.2 ± 143.3	5
			IVR	0.5mg	Monthly		N = 81	NA	+8.7 ± 7.9	NA	-134.7 ± 142.7	
			LASER				N = 82	NA	+0.8 ± 12.3	NA	-103.6 ± 141.7	
Soheilian et al. (2012)	10.5±3.2	NA	IVB	1.25mg	3.1±1.6	24 months	N = 50	+10.5±10	+10.5±13.5	−36 ± 119	−40 ± 133	7
	10.4±2.6	NA	IVB+IVT	1.25mg	2.6±1.5		N = 50	+5±14	+5±13.5	−25 ± 108	−10 ± 145	
				2mg								
	10.5±2.9	NA	LASER	NA	1.0±0.1		N = 50	-1±16.5	+1±17	−11 ± 78	6 ± 86	
Synek et al. (2011)	NA	NA	IVB	1.25mg	3	24 weeks	N = 30	+10±10	NA	–94 ± 170	NA	7
	NA	NA	IVB+IVT	1.25mg	3		N = 30	+10±10	NA	–93 ± 124	NA	
				2mg	1							
DRCR et al. (2010)	16±3.32	89%	LASER	NA	NA	3 years;	N = 293	NA	+3 ± 13	NA	−102 ± 151	7
	18±3.06	92%	IVR+LASER	0.5mg	2 (0–4)		N = 187	NA	+9 ± 11	NA	−131 ± 129	
	17±3.32	89%	IVT+LASER	4mg	1 (0–2)		N = 186	NA	+4 ± 13	NA	−127 ± 140	
Mitchell et al. (2011)	15.23±9.91	88.8%	IVR	0.5mg	7.0±2.81	12 months	N = 116	NA	+6.1 ± 6.43	NA	−118.7 ± 115.07	6
	14.62±9.84	86.4%	IVR+LASER	0.5mg	6.8±2.95		N = 118	NA	+5.9 ± 7.92	NA	−128.3 ± 114.34	
	12.93±9.02	87.4%	LASER	NA	≥3 months		N = 111	NA	+0.8 ± 8.56	NA	−61.3 ± 132.29	
Nguyen et al. (2010)	NA	NA	IVR	0.5mg	4	2 years	N = 42	+7.24 ± 4.46	+6.61 ± 5.58	NA	NA	5
	NA	NA	LASER	NA	NA		N = 42	−0.43 ± 4.45	+2.39 ± 4.0	NA	NA	
	NA	NA	IVR+LASER	0.5mg	2		N = 42	+3.8 ± 4.04	+4.81 ± 5.16	NA	NA	
Michaelides et al. (2010)	NA	89.5%	LASER	NA	3	12 months	N = 38	NA	NA	NA	-68 ± 171	5
	NA	90.5%	IVB	1,25mg	9		N = 42	NA	NA	NA	-130 ± 122	
Massin et al. (2010)	14.2±4.44	97.1%	IVR	0.3 or 0.5mg	10.2±2.5	12 months	N = 102	NA	+10.3 ± 9.1	NA	-194.2 ± 135.1	7
	15.1±6.63	98%	Placebo	NA	NA		N = 49	NA	-1.4 ± 14.2	NA	-48.4 ± 153.4	
Ahmadieh et al. (2008)	NA	NA	IVB	1.25mg	3	24 weeks	N = 41	+9 ± 16.64	NA	−95.7 ± 172.5	NA	6
	NA	NA	IVB+IVT	1.25mg	3		N = 37	+10.5 ± 13.50	NA	−92.1 ± 125.3	NA	
				2mg	1							
	NA	NA	Placebo	NA	NA		N = 37	+1.5 ± 16.50	NA	34.9 ± 63.9	NA	
Pappas et al. (2008)	NA	NA	IVT+LASER	4mg	NA	6 months	N = 35	NA	NA	-176.14 ± 46	NA	5
	NA	NA	IVB	1.25mg	NA		N = 27	NA	NA	-87.38 ± 21	NA	
Lam et al. (2007)	NA	NA	IVT	4mg	1	6 months	N = 38	-0.7 ± 10.7	NA	NA	NA	5
	NA	NA	IVT+LASER	4mg	1		N = 36	-1.1 ± 10.8	NA	NA	NA	
					1							
	NA	NA	LASER	NA	1		N = 37	-1.6 ± 11.5	NA	NA	NA	
Audren et al. (2006)	14.4±8.9	NA	IVT	4mg	1	6 months	N = 17	+6.9 ± 10.7	NA	NA	NA	5
			Placebo	NA	NA		N = 17	-2.6 ± 7.8	NA	NA	NA	

BCVA, mean change in best corrected visual acuity; CMT, mean change in central macular thickness; IVA, intravitreal aflibercept; IVB, intravitreal bevacizumab; IVR, intravitreal ranibizumab; LASER, laser, macular laser, grid laser and focal/grid laser; DDSI, dexamethasone implant; IVT, intravitreal triamcinolone; NA, No available.

### Data Abstraction and Quality Assessment

We assessed the risk of bias in each study using the Cochrane Collaboration’s tool and evaluated the risk of bias categories based on the following items: (1) sequence generation, (2) allocation concealment, (3) blinding of participants and personnel, (4) incomplete outcomes data, (5) selective reporting, and (6) other bias. We also reassessed the bias using the modified Jadad scale. Randomization, concealment of allocation, double blinding, and withdrawals and dropouts were evaluated with a total of 7 points.

Characteristics of studies, such as author, year of publication, duration and type of diabetes, dosage and number of each intervention, follow-up time, and number of eyes in each intervention were recorded exactly. The details of the efficacy outcome, including the mean change of BCVA and CMT in 6 months and 12 months from the baseline, were captured respectively. When extracting the BCVA data, we converted the logarithm of the minimal angle of resolution (log MAR) into ETDRS letters form.

### Statistical Analysis

We performed the multi-treatment meta-analysis within a Bayesian framework by using the Markov Chain Monte Carlo simulation [24]. All data were analyzed by using the Aggregate Data Drug Information System (ADDIS) v1.16.5 (Drugis, Groningen, NL). Forest plots were made in R software (version 3.2.3) with the R2winBUGS package. Statistical heterogeneity was assessed by the I^2^ using the Higgins–Thompson method [25]: < 25% was no heterogeneity, 25–50% was low heterogeneity, 50–75% was moderate heterogeneity, and > 75% was high heterogeneity.

Node-splitting [26] and pair-wise meta-analyses were used to evaluate the inconsistency of direct comparisons in indirect evidences in the network meta-analysis. The direct and indirect evidences in accordance in the split node were analyzed in a node-splitting assessment. P < 0.05 was considered as significant heterogeneity. The efficacy of the intervention was assessed by the odds ratio (OR) with 95% credibility interval (CI). If 1.0 was not included in 95% CI, the results were considered statistically significant. We calculated the rank’s possibility and ranked the outcome of different inventions according to the estimated effect size.

## Results

### Literature Search

**Fig 1:**
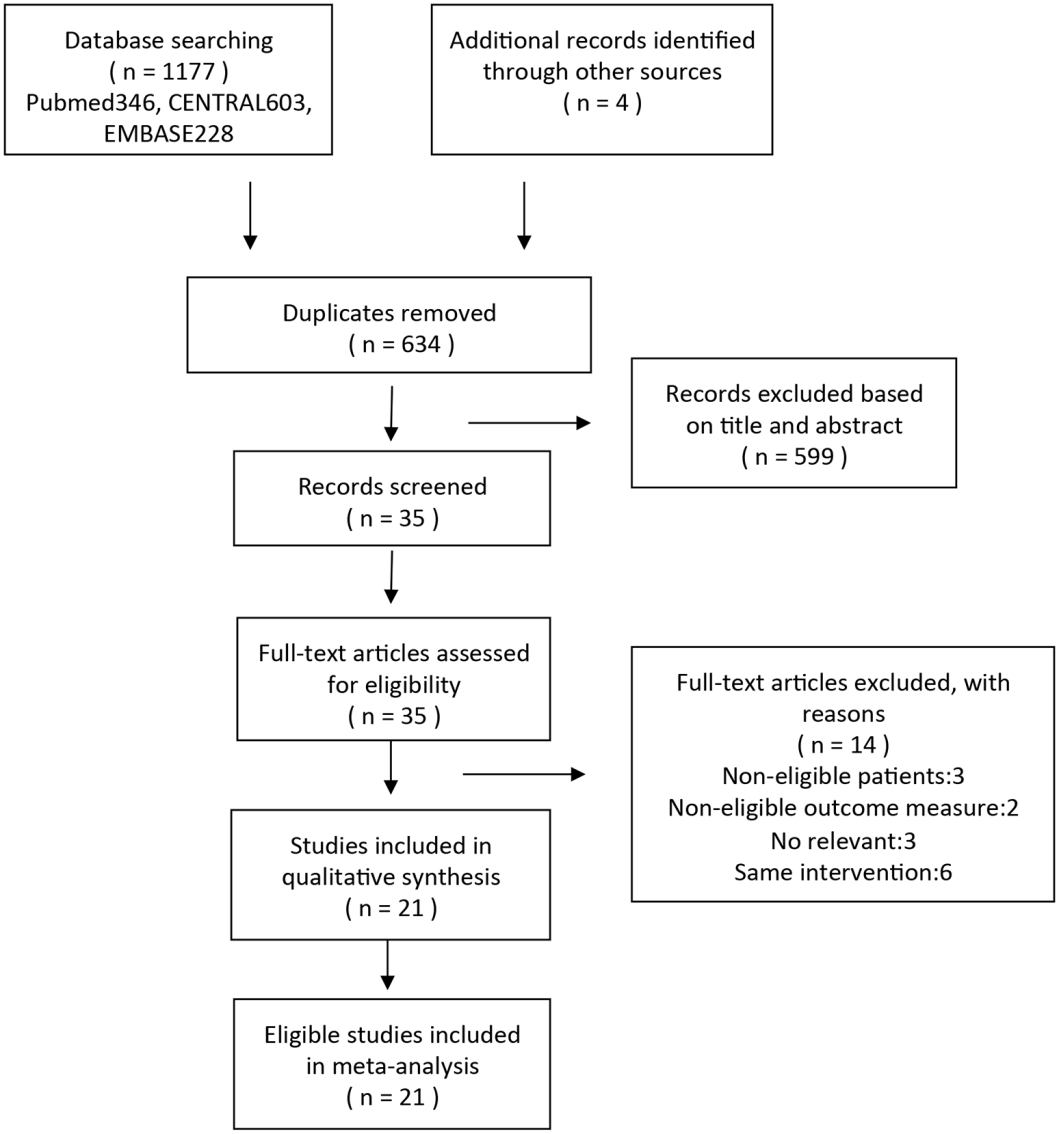
Flow chart indicating the selection process for this network meta-analysis.

We identified 1181 articles (346 in PubMed, 603 in Embase, 228 in CENTRAL, and 4 additional records through other sources) in the initial search of all databases. These articles were restricted to human subjects and RCTs before August 2015. Except 547 studies that were retrieved for duplicates, there were 634 studies. Based on the titles and abstracts, 599 of them were removed. Of the remained 35 studies, 14 articles were excluded, because the patients in 3 trials and the outcome measures in 2 trials did not meet the inclusion criteria, the interventions in 6 trials were same, and the study types in 3 trials were not relevant with our meta-analysis. A total of 21 trials were identified and were eligible for this net meta-analysis (Fig 1)[27–47].

### Study Characteristics

**Fig 2:**
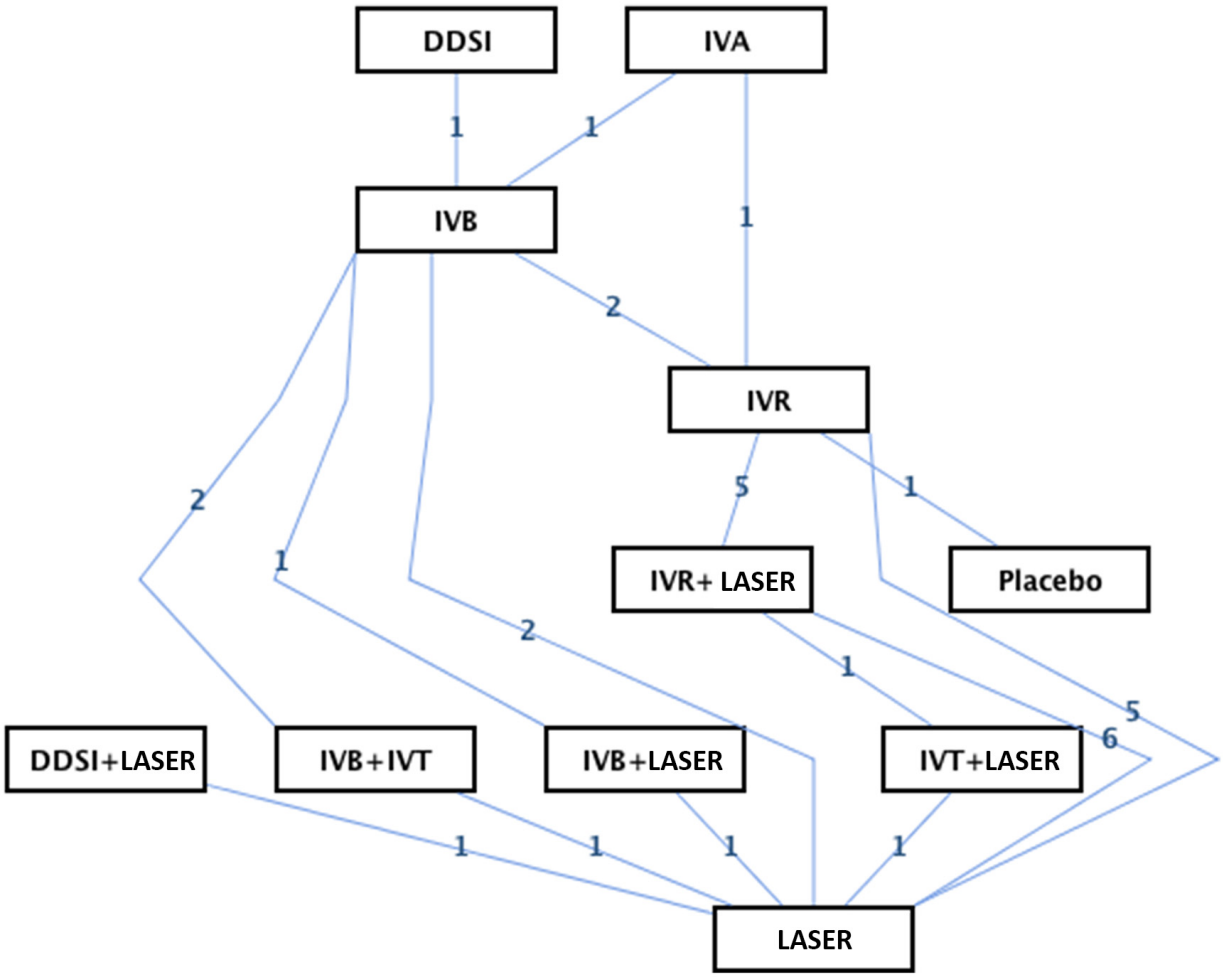
Network of eligible comparisons for the meta-analysis.

The eligible 21 trials covered 11 different interventions in the treatment of DME and resulted in 16 theoretical comparisons for each of the primary outcomes. We conducted a network of eligible comparisons for the multiple-treatment meta-analysis (Fig 2) and compared the primary endpoints of BCVA and CMT (Tables 2 and 3).

**Table 2:** Network meta-analysis results in BCVA (upper part) and CMT (lower part) at 6 months.

IVB	1.30 (-3.09, 5.54)	4.31 (-2.39, 10.51)	-1.26 (-7.43, 5.43)	1.30 (-5.08, 8.05)	3.41 (-4.21, 10.49)	4.48 (-4.36, 12.59)	**5.69 (1.45, 10.70)**	**10.88 (4.08, 17.58)**
-6.47 (-72.54, 62.87)	IVB+IVT	2.82 (-4.42, 10.10)	-2.73 (-9.34, 4.83)	-0.16 (-6.73, 7.36)	1.93 (-5.70, 9.57)	3.26 (-6.05, 11.94)	4.31 (-0.81, 10.40)	**9.62 (2.69, 16.67)**
-	-	IVBLASER	-5.61 (-12.55, 2.31)	-3.02 (-10.08, 4.93)	-0.86 (-9.55, 7.55)	0.15 (-9.70, 9.59)	1.39 (-4.33, 8.07)	6.74 (-2.12, 15.42)
42.28 (-106.95, 193.39)	49.32 (-105.51, 197.57)	-	IVR	2.65 (-1.98, 6.92)	4.54 (-3.29, 11.91)	5.89 (-3.46, 13.93)	**7.01 (2.56, 11.39)**	**12.23 (3.57, 20.22)**
28.80 (-120.93, 176.05)	34.23 (-115.90, 183.08)	-	-14.67 (-123.21, 92.73)	IVR+LASER	1.91 (-5.97, 9.49)	3.27 (-5.99, 11.58)	4.41 (0.04, 8.89)	9.60 (0.82, 17.75)
-	-	-	-	-	IVT	1.20 (-5.98, 8.22)	2.49 (-3.61, 9.08)	7.57 (0.60, 14.05)
87.93 (-15.39, 197.99)	95.31 (-30.93, 225.58)	-	46.60 (-141.00, 226.56)	59.41 (-126.36, 243.64)	-	IVT+LASER	1.17 (-5.85, 9.26)	6.43 (-2.80, 15.28)
-23.27 (-121.84, 81.43)	-16.45 (-117.29, 86.00)	-	-66.32 (-176.81, 46.22)	-51.41 (-157.00, 63.95)	-	-111.34 (-254.61, 37.93)	LASER	5.10 (-2.19, 12.07)
**-131.82 (-231.95, -28.49)**	**-126.16 (-227.11, -20.62)**	-	-174.45 (-349.45, 3.79)	-159.11 (-333.08, 18.42)	-	**-220.07 (-368.98, -71.10)**	-108.67 (-246.74, 28.57)	Placebo

IVB, intravitreal bevacizumab; IVR, intravitreal ranibizumab; LASER, laser, macular laser, grid laser and focal/grid laser; IVT, intravitreal triamcinolone.

**Table 3:** Network meta-analysis results in BCVA (upper part) and CMT (lower part) at 12 months.

DDSI	0.41 (-6.98, 8.14)	**-7.07 (-13.77, -0.27)**	-3.38 (-9.45, 2.45)	0.53 (-7.14, 8.14)	-1.07 (-8.11, 6.43)	-4.97 (-11.17, 1.35)	-4.09 (-10.39, 2.33)	0.56 (-6.44, 7.72)	1.20 (-5.07, 7.84)	6.74 (-1.38, 14.99)
-	DDSI+LASER	**-7.38 (-12.97, -2.40)**	**-3.72 (-8.71, 0.62)**	0.26 (-6.73, 6.32)	-1.36 (-7.24, 4.23)	**-5.35 (-9.74, -1.10)**	-**4.46 (-8.88, -0.33)**	0.17 (-5.01, 5.28)	0.81 (-3.16, 4.73)	6.36 (-0.61, 12.96)
-	**110.83 (35.27, 190.25)**	IVA	3.71 (0.36, 6.68)	**7.60 (1.73, 13.14)**	**5.99 (1.24, 11.17)**	2.07 (-0.97, 5.33)	2.93 (-0.34, 6.57)	**7.55 (3.23, 12.37)**	**8.19 (5.07, 11.96)**	**13.84 (7.84, 19.71)**
-	56.20 (-9.79, 124.73)	**-54.65 (-104.84, -3.26)**	IVB	3.95 (-0.91, 8.61)	2.32 (-1.58, 6.76)	**-1.67 (-3.35, 0.74)**	-0.80 (-2.87, 2.08)	3.85 (0.35, 8.19)	**4.47 (2.56, 7.28)**	**10.11 (4.76, 15.79)**
-	31.16 (-49.84, 115.46)	**-79.38 (-154.61, -4.14)**	-25.14 (-82.54, 32.81)	IVB+IVT	-1.60 (-7.72, 4.97)	**-5.56 (-10.41, -0.34)**	**-4.69 (-9.79, 0.75)**	-0.10 (-5.67, 6.39)	0.60 (-4.43, 6.00)	6.25 (-1.07, 13.46)
-	-	-	-	-	IVB+LASER	**-3.96 (-8.20, 0.13)**	-3.09 (-7.41, 1.09)	1.55 (-3.65, 6.69)	2.20 (-1.87, 6.30)	**7.84 (1.01, 14.18)**
-	**74.43 (12.00, 137.89)**	-36.39 (-87.43, 13.00)	18.24 (-13.25, 47.58)	43.14 (-17.96, 103.47)	-	IVR	0.87 (-0.65, 2.45)	**5.48 (2.18, 9.08)**	**6.14 (4.74, 7.84)**	**11.76 (6.55, 16.73)**
-	**83.42 (20.92, 146.51)**	-27.30 (-84.80, 28.17)	27.00 (-12.56, 64.73)	52.15 (-10.76, 114.94)	-	9.00 (-18.60, 38.21)	IVR+LASER	**4.61 (1.32, 8.02)**	**5.28 (3.80, 6.90)**	**10.93 (5.43, 16.03)**
-	63.27 (-11.16, 141.16)	-46.93 (-119.78, 23.15)	7.55 (-51.67, 64.19)	32.69 (-45.99, 108.50)	-	-10.83 (-65.55, 43.23)	-19.72 (-70.09, 30.24)	IVT+LASER	0.66 (-2.64, 3.95)	6.25 (-0.15, 12.30)
-	22.23 (-34.26, 80.08)	**-88.50 (-143.87, -35.10)**	**-34.08 (-67.36, -1.11)**	-9.18 (-68.11, 49.90)	-	**-52.11 (-77.09, -26.21)**	**-61.14 (-87.68, -34.75)**	-41.07 (-91.20, 9.57)	LASER	5.59 (0.08, 10.71)
-	-71.76 (-161.34, 21.75)	**-182.93 (-268.84, -96.95)**	-**128.15 (-202.57, -50.77)**	-**103.81 (-193.32, -9.21)**	-	**-145.96 (-213.86, -75.51)**	**-155.43 (-227.86, -78.77)**	**-135.36 (-222.74, -46.31)**	**-94.01 (-165.76, -18.93)**	Placebo

IVA, intravitreal aflibercept; IVB, intravitreal bevacizumab; IVR, intravitreal ranibizumab; LASER, laser, macular laser, grid laser and focal/grid laser; DDSI, dexamethasone implant; IVT, intravitreal triamcinolone.

Characteristics of the 21 included studies are presented in Table 1. The 21 eligible RCTs contained a total of 4307 eyes, including 428 eyes with 6-month follow-up and 3879 eyes with 12-month follow-up. The data of BCVA and CMT in 6 and 12 months were recorded respectively for the network meta-analysis.

### Risk of Bias

**Fig 3:**
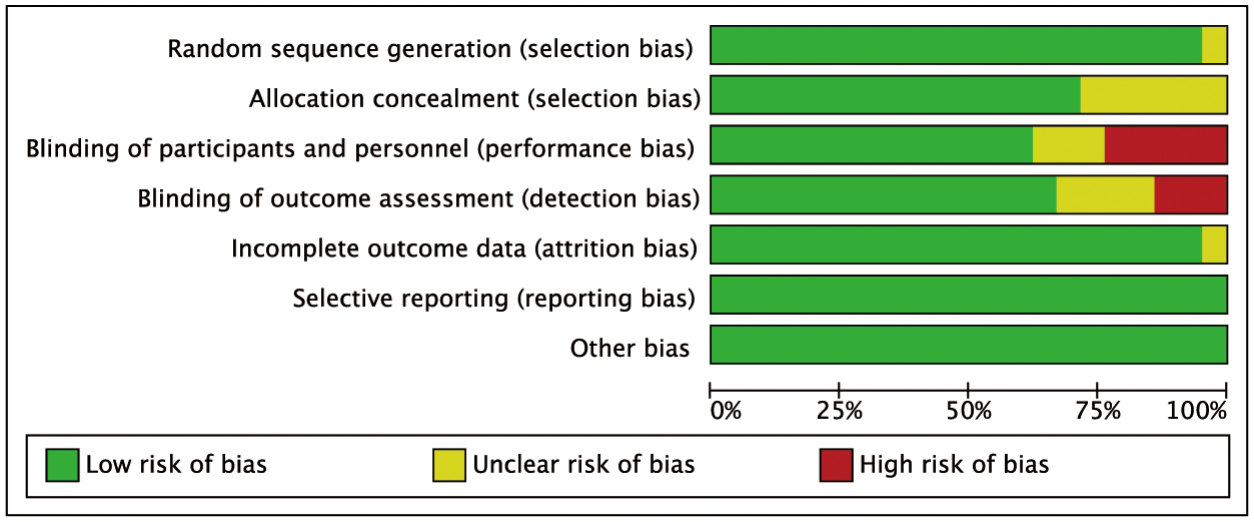
Risk of bias graph.

**Fig 4:**
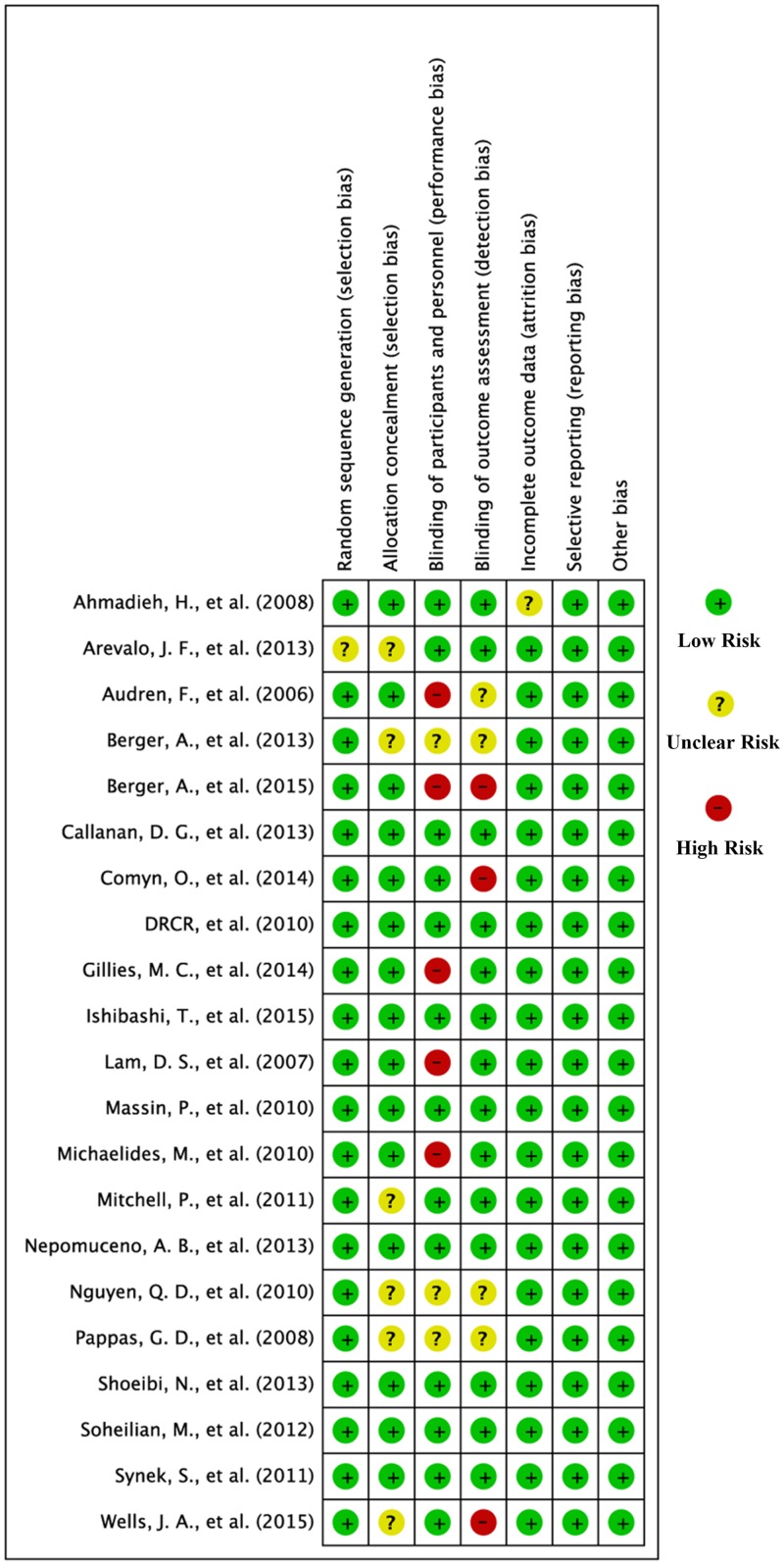
Risk of bias summary.

The biases of the 21 included studies were assessed by using the Cochrane Collaboration’s tool as shown in Figs 3 and 4. One of the trials did not describe the method used for generating the allocation sequence [36]; six not for allocation concealment [27, 29, 36, 39, 40, 44]; three not for blinding of participants and personnel [29, 40, 44]; four not for blinding of outcome assessment [29, 40, 44, 47]; one not for incomplete outcome data [45]. Therefore, the risk of bias was considered unclear. In all, five trials performed a high risk of bias in blinding of participants and personnel [30, 35, 41, 46, 47], and three studies featured a high risk of outcome assessment [27, 31, 35].

The modified Jadad scale of 21 trials is listed in Table 1, showing similar results to the bias using the Cochrane Collaboration’s tool.

### Visual Acuity

Since BCVA is the primary efficacy and progress indicator, and CMT is an important anatomical indicator in treating DME, data of BCVA and CMT were analyzed. For the baseline BCVA and CMT were not strictly matched, the mean change of BCVA and CMT rather than BCVA and CMT themselves was analyzed as the primary outcome (Tables 2 and 3).

**Fig 5:**
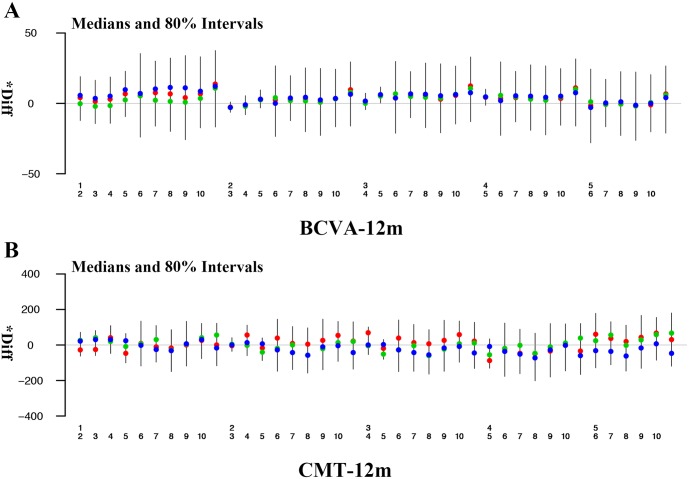
Forest plots for BCVA and CMT in 12 months. Abbreviations: 1, IVA; 2, IVB; 3, IVR; 4, IVR+LASER; 5, LASER; 6, DDSI; 7, IVB+IVT; 8, DDSI+LASER; 9, IVB+LASER; 10, IVT+LASER; 11, Placebo.

The results of mean BCVA changes in 6 months from baseline showed that intravitreal ranibizumab (IVR) 7.01 (2.56 to 11.39) was best, followed by intravitreal bevacizumab (IVB) 5.69 (1.45 to 10.70) and IVR plus LASER 4.41 (0.04 to 8.89) when compared with LASER alone (Table 2). Both IVR and IVB were significantly superior to LASER alone. All comparisons showed no significant heterogeneity (all p>0.05, S1 Table). In 12 months, intravitreal aflibercept (IVA) 8.19 (5.07 to 11.96) was best, followed by IVR 6.14 (4.74 to 7.84) and IVR plus LASER 5.28 (3.80 to 6.90) when compared with LASER alone (Table 3, Fig 5). All of them were significantly superior to LASER alone. However, there was significant heterogeneity in the comparison between IVB and LASER (p = 0.04, S1 Table). IVR and IVA were superior to other therapies on the basis of mean BCVA changes from baseline in 6 and 12 months. Ranking based on simulations is available in the Supplemental Materials and Methods (See S2 Table).

### Central Macular Thickness

Analyses of mean CMT changes in 6 months from baseline showed that intravitreal triamcinolone (IVT) plus LASER -111.34 (-254.61 to 37.93) was best, followed by IVR -66.32 (-176.81 to 46.22) and IVR plus LASER -51.41 (-157.00 to 63.95), when compared with LASER alone (Table 2). None of them was statistically superior to LASER alone. In 12 months, IVA -110.83 (-190.25 to -35.27) was best, followed by IVR plus LASER -83.42 (-146.51 to -20.92) and IVR -74.43 (-137.89 to -12.00), when compared with DDSI+ LASER (Table 3, Fig 5). And all of IVA, IVR and IVR plus LASER were significantly superior to DDSI+LASER. All comparisons showed no significant heterogeneity in 12 months (all p>0.05, S1 Table). IVT plus LASER was better than IVR and IVR plus LASER on the basis of mean CMT changes from baseline in 6 months. Meanwhile, IVA was better than IVR and IVR plus LASER on the basis of mean CMT changes from baseline in 12 months. Ranking based on simulations is available in the Supplemental Materials and Methods (See S2 Table).

### Adverse Events

Of the 21 trials with 4307 eyes involved, the most common ocular AEs were IOP increase and cataract progress. In 13 trials, IOP increase was reported, and the incidence of IOP increase was 47.7% in the IVT plus LASER group [43, 46], 7.6% in the IVR plus LASER group [29, 39, 43], 7.5% in the IVB group [27, 30, 41], 7.2% in the IVA group [27], and 4.8% in the IVR group [27, 29, 39]. Furthermore, cataract progression was reported in 8 trials, and the rate was 26.9% in the IVT plus LASER group [43], 8% in the IVR group [29], 6.5% in the IVB group [30, 33, 37], 6.3% in the IVR plus LASER group [28, 43], and 0.5% in the IVA group [27]. The groups with administration of steroids had a higher incidence of IOP increase and cataract progress than the groups with use of VEGF inhibitors in the treatment of DME.

Hypertension, angina, and myocardial infarction were reported as systematic AEs potentially related to VEGF inhibition. The incidence of hypertension was reported in 8 trials, being 12.5% in the IVA group [27], 7.6% in the IVR group [27–29, 33, 39, 42], 6.2% in the IVB group [27, 30, 41], and 4.9% in the IVR plus LASER group [27, 28]. The rate of angina was 0.8% in the IVR group [28]. Myocardial infarction was reported in 3 trials, with a rate of 1.9% in the IVA group, 0.5% in the IVB group [27], and 1.2% in the IVR group [27, 39, 42].

### Consistency of Network-model and Sensitivity Analysis

Based on direct versus indirect evidences, we compared the effect estimate using the node-splitting and pair-wise meta-analysis (S1 Table). No inconsistencies were observed. We performed the sensitivity analysis of comparison of random and fixed effects model that was more accurate. The outcome was not changed (S3 Table). Those data suggested that our model was very robust.

## Discussion

In this network meta-analysis concerning 21 trials and 4307 eyes, we reviewed published information and analyzed the efficacy and safety of therapeutic approaches in the management of DME. It was indicated that the most efficacious treatment was IVA based on the mean BCVA and CMT changes in 12 months, followed by IVR. This was similar to a recent publication showing that greater BCVA improvement was seen with aflibercept than with bevacizumab or ranibizumab [27].

Meanwhile, we found that IVT combined with LASER was best in decreasing CMT in 6 months with no statistical significance. One of the reasons is that IVT plus LASER might have a stronger anti-inflammatory and anti-angiogenic effect in early stage after injection. This only affects the CMT decrease, but has no impact on BCVA improvement [19]. The other reason may be the bias of 3 trials concerning IVT plus LASER [43, 44, 46]. It was a median risk for no description of allocation concealment, blinding of participants and personnel, and blinding of outcome assessment in the report of Pappas et al, but was a high risk of blinding of participants and personnel according to the results of Lam et al. Moreover, there was no trial reporting CMT data related to the IVA treatment in 6 months. Therefore, clinicians should notice that IVT combined with LASER might have benefits in the early stage after injection for decreasing CMT.

There was no statistically significant difference between IVR and IVR plus LASER in either BCVA improvement or CMT decrease during the follow-up period. Another finding was that LASER was superior to placebo only in decreasing CMT in 12 months, suggesting that the role of LASER in the treatment of DME should be reappraised. Meanwhile, 13 trials involved laser treatment in our study, including macular laser (4 trials), grid laser (3 trials), and focal/grid laser (6 trials). Although focal and grid laser photocoagulations belong to macular laser treatment [48], there may be heterogeneity in these two macular laser treatments. Our meta-analysis was based on an assumption that the LASER therapies were same, and clinicians should pay attention to this.

Among ocular AEs, high IOP occurred more frequently in the IVT plus LASER group (47.7%) than the anti-VEGF groups (4.8%-7.2%), while cataract progression was also mostly found in the IVT plus LASER (47.7%) group than the anti-VEGF groups (4.8%-7.2%). Those data were consistent with the notion that there were more ocular AEs in the steroids treatment groups [49]. The incidence of hypertension and myocardial infarction in the anti-VEGF groups was higher than the steroids groups. Diabetic patients are known to be at a higher risk of cardiovascular comorbidities, which are susceptible to systemic complications in addition to DME. The data were in keeping with previously reported AEs occurring with anti-VEGF treatment [11].

Our network meta-analysis focused on the drugs commonly used in patients with DME, and the results of this work may be important for clinical treatment. However, there are also limitations that need to be taken into account. The challenge in the network meta-analysis is that characteristics of the included 21 trials could not be matched, such as the duration of diabetics, the dosage and number of each intervention and the type of laser treatment. Moreover, the eligible 21 trials did not present the outcomes of patients in different types of diabetes separately, so it remains unknown whether any specific type of diabetes could alter the outcomes. The definition and details of AEs were not always reported in each involved study, so that it was not possible to assess the exact incidence of AEs. Therefore, the data available can only indicate the relative safety of every intervention for DME. To more accurately assess the efficacy of these treatments, additional high-quality RCTs will be necessary.

## Conclusions

Our analysis confirms that intravitreal aflibercept is most favorable for BCVA improvement and CMT decrease compared with other current therapies in the management of DME within 12 months. VEGF inhibitors for DME should be used with caution due to systematic AEs. Combined IVT with LASER has a stronger efficacy in decreasing CMT than the other interventions in the early stage after injection. More high-quality randomized controlled trials will be necessary.

## Supporting Information

S1 PRISMA Checklist (DOCX)

S1 Table Node-splitting and pair-wise meta-analysis. (DOCX)

S2 Table Ranking based on simulations. (DOCX)

S3 Table Sensitivity analysis for included studies of DME. (DOCX)
