# Supplementary material for: The Efficacy and Safety of Current Treatments in Diabetic Macular Edema: A Systematic Review and Network Meta-Analysis
Source: PLoS One. 2016 Jul 19;11(7):e0159553. doi: 10.1371/journal.pone.0159553 (PMC4951132; doi:10.1371/journal.pone.0159553)
Supplement: S1 Table — (DOCX) [file pone.0159553.s002.docx]

**S1 Table.**

| **Name** | **Direct Effect** | **Indirect Effect** | **Overall** | **P-Value** | **Pair-wise meta-analyses** | **Heterogeneity** | **N of trials** |
| --- | --- | --- | --- | --- | --- | --- | --- |
| **BCVA (6-month)** |  |  |  |  |  |  |  |
| IVB, LASER | -6.35 (-12.19, -1.66) | -0.82 (-15.41, 12.63) | -5.69 (-10.70, -1.45) | 0.39 | -7.43 (-14.69, -0.17) | I2 = 79.0% | 2 |
| IVB, Placebo | -8.83 (-17.80, 0.70) | -15.05 (-27.58, -2.16) | -10.88 (-17.58, -4.08) | 0.41 | -7.50 (-14.86, -0.14) | I2 = NA | 1 |
| IVB+IVT, LASER | -7.71 (-15.76, 0.19) | -1.74 (-8.08, 5.01) | -4.31 (-10.40, 0.81) | 0.18 | -6.00(-12.46, 0.46) | I2 = NA | 1 |
| IVB+IVT, Placebo | -8.08 (-16.92, 0.52) | -13.75 (-27.32, -1.33) | -9.62 (-16.67, -2.69) | 0.42 | -9.00 (-15.87, -2.13) | I2 = NA | 1 |
| IVT, LASER | -0.57 (-8.02, 6.25) | -6.80 (-19.04, 5.71) | -2.49 (-9.08, 3.61) | 0.35 | -0.90 (-5.93, 4.13) | I2 = NA | 1 |
| IVT, Placebo | -9.42 (-18.36, -0.73) | -3.61 (-15.79, 9.33) | -7.57 (-14.05, -0.60) | 0.38 | -9.50 (-15.79, -3.21) | I2 = NA | 1 |
| **BCVA (12-month)** |  |  |  |  |  |  |  |
| IVB, IVR | 2.58 (0.53, 4.20) | -1.85 (-5.68, 2.06) | 1.67 (-0.74, 3.35) | 0.06 | 2.50 (1.11, 3.89) | I2 = 51.2% | 2 |
| IVB, LASER | -7.44 (-10.85, -4.08) | -2.93 (-5.61, -0.99) | -4.47 (-7.28, -2.56) | 0.04 | -7.36 (-10.51, -4.22) | I2 = 0.0% | 2 |
| IVB+IVT, LASER | -4.32 (-10.94, 2.79) | 1.37 (-4.34, 6.43) | -0.60 (-6.00, 4.43) | 0.14 | -4.00 (-10.70, 2.70) | I2 = NA | 1 |
| IVR, IVR+LASER | -0.81 (-2.44, 0.91) | -0.13 (-4.27, 3.60) | -0.87 (-2.45, 0.65) | 0.74 | -0.66 (-1.69, 0.37) | I2 = 0.0% | 5 |
| IVR, LASER | -5.56 (-7.15, -4.15) | -8.03 (-10.64, -5.65) | -6.14 (-7.84, -4.74) | 0.08 | -5.62 (-7.03, -4.22) | I2 = 40.0% | 5 |
| **CMT (12-month)** |  |  |  |  |  |  |  |
| IVB, IVR | -25.16 (-63.83, 10.43) | 4.75 (-52.15, 65.27) | -18.24 (-47.58, 13.25) | 0.35 | -26.34 (-61.46, 8.79) | I2 = 84.8% | 2 |
| IVB, LASER | 51.08 (-0.80, 105.11) | 21.35 (-27.65, 66.84) | 34.08 (1.11, 67.36) | 0.37 | 51.49 (13.00, 89.99) | I2 = 32.6% | 2 |
| IVB+IVT, LASER | 15.28 (-57.11, 87.03) | -1.47 (-80.19, 72.66) | 9.18 (-49.90, 68.11) | 0.70 | 16.00 (-36.93, 68.93) | I2 = NA | 1 |
| IVR, IVR+LASER | -15.78 (-42.97, 11.65) | 27.47 (-24.58, 78.08) | -9.00 (-38.21, 18.60) | 0.12 | -17.26 (-35.88, 1.36) | I2 = 0% | 4 |
| IVR, LASER | 54.15 (24.40, 79.99) | 43.06 (4.39, 89.12) | 52.11 (26.21, 77.09) | 0.66 | 55.18 (36.51, 73.86) | I2 = 6.4% | 5 |

Node-splitting and pair-wise meta-analysis.

BCVA, mean change in best corrected visual acuity; IVB, intravitreal bevacizumab; LASER, laser, macular laser, grid laser and focal/grid laser; IVT, intravitreal triamcinolone; IVR, intravitreal ranibizumab; CMT, mean change in central macular thickness.
