# Supplementary material for: The Efficacy and Safety of Current Treatments in Diabetic Macular Edema: A Systematic Review and Network Meta-Analysis
Source: PLoS One. 2016 Jul 19;11(7):e0159553. doi: 10.1371/journal.pone.0159553 (PMC4951132; doi:10.1371/journal.pone.0159553)
Supplement: S2 Table — (DOCX) [file pone.0159553.s003.docx]

**S2 Table.**

BCVA (6-month)

| **Drug** | **Rank 1** | **Rank 2** | **Rank 3** | **Rank 4** | **Rank 5** | **Rank 6** | **Rank 7** | **Rank 8** | **Rank 9** |
| --- | --- | --- | --- | --- | --- | --- | --- | --- | --- |
| IVB | 0.20 | 0.34 | 0.24 | 0.14 | 0.06 | 0.03 | 0.01 | 0.00 | 0.00 |
| IVB+IVT | 0.10 | 0.14 | 0.21 | 0.23 | 0.16 | 0.09 | 0.04 | 0.02 | 0.00 |
| IVB+LASER | 0.02 | 0.03 | 0.05 | 0.09 | 0.19 | 0.20 | 0.22 | 0.17 | 0.03 |
| IVR | 0.58 | 0.18 | 0.14 | 0.05 | 0.03 | 0.01 | 0.01 | 0.00 | 0.00 |
| IVR+LASER | 0.03 | 0.20 | 0.23 | 0.26 | 0.15 | 0.08 | 0.03 | 0.01 | 0.00 |
| IVT | 0.04 | 0.07 | 0.08 | 0.13 | 0.22 | 0.19 | 0.15 | 0.11 | 0.01 |
| IVT+LASER | 0.04 | 0.04 | 0.06 | 0.09 | 0.13 | 0.20 | 0.17 | 0.23 | 0.05 |
| LASER | 0.00 | 0.00 | 0.00 | 0.01 | 0.05 | 0.18 | 0.34 | 0.38 | 0.04 |
| Placebo | 0.00 | 0.00 | 0.00 | 0.00 | 0.01 | 0.01 | 0.03 | 0.08 | 0.86 |

BCVA (12-month)

| **Drug** | **Rank 1** | **Rank 2** | **Rank 3** | **Rank 4** | **Rank 5** | **Rank 6** | **Rank 7** | **Rank 8** | **Rank 9** | **Rank 10** | **Rank 11** |
| --- | --- | --- | --- | --- | --- | --- | --- | --- | --- | --- | --- |
| DDSI | 0.02 | 0.04 | 0.03 | 0.05 | 0.14 | 0.16 | 0.12 | 0.10 | 0.11 | 0.20 | 0.04 |
| DDSI+LASER | 0.00 | 0.00 | 0.01 | 0.03 | 0.11 | 0.17 | 0.18 | 0.17 | 0.15 | 0.15 | 0.02 |
| IVA | 0.89 | 0.06 | 0.03 | 0.01 | 0.00 | 0.00 | 0.00 | 0.00 | 0.00 | 0.00 | 0.00 |
| IVB | 0.00 | 0.05 | 0.16 | 0.54 | 0.21 | 0.04 | 0.00 | 0.00 | 0.00 | 0.00 | 0.00 |
| IVB+IVT | 0.00 | 0.01 | 0.01 | 0.03 | 0.10 | 0.15 | 0.15 | 0.13 | 0.15 | 0.21 | 0.03 |
| IVB+LASER | 0.00 | 0.02 | 0.03 | 0.07 | 0.25 | 0.25 | 0.16 | 0.10 | 0.07 | 0.05 | 0.01 |
| IVR | 0.06 | 0.71 | 0.18 | 0.04 | 0.01 | 0.00 | 0.00 | 0.00 | 0.00 | 0.00 | 0.00 |
| IVR+LASER | 0.01 | 0.10 | 0.55 | 0.23 | 0.08 | 0.02 | 0.00 | 0.00 | 0.00 | 0.00 | 0.00 |
| IVT+LASER | 0.00 | 0.00 | 0.00 | 0.01 | 0.08 | 0.16 | 0.22 | 0.19 | 0.17 | 0.15 | 0.01 |
| LASER | 0.00 | 0.00 | 0.00 | 0.00 | 0.00 | 0.03 | 0.15 | 0.31 | 0.34 | 0.16 | 0.00 |
| Placebo | 0.00 | 0.00 | 0.00 | 0.00 | 0.00 | 0.00 | 0.01 | 0.01 | 0.02 | 0.08 | 0.88 |

CMT (6-month)

| **Drug** | **Rank 1** | **Rank 2** | **Rank 3** | **Rank 4** | **Rank 5** | **Rank 6** | **Rank 7** |
| --- | --- | --- | --- | --- | --- | --- | --- |
| IVB | 0.00 | 0.12 | 0.26 | 0.37 | 0.15 | 0.10 | 0.01 |
| IVB+IVT | 0.01 | 0.20 | 0.35 | 0.25 | 0.12 | 0.06 | 0.01 |
| IVR | 0.01 | 0.03 | 0.05 | 0.08 | 0.21 | 0.47 | 0.14 |
| IVR+LASER | 0.02 | 0.05 | 0.10 | 0.12 | 0.39 | 0.25 | 0.07 |
| IVT+LASER | 0.00 | 0.01 | 0.01 | 0.02 | 0.08 | 0.11 | 0.76 |
| LASER | 0.02 | 0.55 | 0.21 | 0.15 | 0.05 | 0.01 | 0.00 |
| Placebo | 0.94 | 0.03 | 0.02 | 0.01 | 0.00 | 0.00 | 0.00 |

CMT (12-month)

| **Drug** | **Rank 1** | **Rank 2** | **Rank 3** | **Rank 4** | **Rank 5** | **Rank 6** | **Rank 7** | **Rank 8** | **Rank 9** |
| --- | --- | --- | --- | --- | --- | --- | --- | --- | --- |
| DDSI+LASER | 0.06 | 0.64 | 0.17 | 0.08 | 0.03 | 0.01 | 0.01 | 0.00 | 0.00 |
| IVA | 0.00 | 0.00 | 0.00 | 0.00 | 0.01 | 0.03 | 0.05 | 0.10 | 0.81 |
| IVB | 0.00 | 0.00 | 0.02 | 0.14 | 0.46 | 0.27 | 0.07 | 0.03 | 0.00 |
| IVB+IVT | 0.01 | 0.17 | 0.23 | 0.32 | 0.14 | 0.06 | 0.03 | 0.03 | 0.01 |
| IVR | 0.00 | 0.00 | 0.00 | 0.01 | 0.06 | 0.28 | 0.43 | 0.20 | 0.02 |
| IVR+LASER | 0.00 | 0.00 | 0.00 | 0.01 | 0.03 | 0.09 | 0.26 | 0.51 | 0.11 |
| IVT+LASER | 0.00 | 0.01 | 0.03 | 0.12 | 0.24 | 0.26 | 0.15 | 0.13 | 0.05 |
| LASER | 0.00 | 0.12 | 0.54 | 0.31 | 0.02 | 0.00 | 0.00 | 0.00 | 0.00 |
| Placebo | 0.93 | 0.06 | 0.01 | 0.00 | 0.00 | 0.00 | 0.00 | 0.00 | 0.00 |

Ranking based on simulations.

BCVA, mean change in best corrected visual acuity; IVB, intravitreal bevacizumab; IVT, intravitreal triamcinolone; LASER, laser, macular laser, grid laser and focal/grid laser; IVR, intravitreal ranibizumab; DDSI, dexamethasone implant; IVA, intravitreal aflibercept; CMT, mean change in central macular thickness.
