# Supplementary material for: The Efficacy and Safety of Current Treatments in Diabetic Macular Edema: A Systematic Review and Network Meta-Analysis
Source: PLoS One. 2016 Jul 19;11(7):e0159553. doi: 10.1371/journal.pone.0159553 (PMC4951132; doi:10.1371/journal.pone.0159553)
Supplement: S3 Table — (DOCX) [file pone.0159553.s004.docx]

**S3 Table.**

| **BCVA-12m** | **Totresdev** | **pD** | **DIC** |
| --- | --- | --- | --- |
| Random effects model | 24.45 | 24.1 | 282.0 |
| Fixed effects model | 23.91 | 23.5 | 280.9 |
| **CMT-12m** | **Totresdev** | **pD** | **DIC** |
| Random effects model | 16.44 | 11.7 | 406.0 |
| Fixed effects model | 16.48 | 11.5 | 405.9 |

Sensitivity analysis for included studies of DME.

BCVA, mean change in best corrected visual acuity; CMT, mean change in central macular thickness.
